# Recent trends in self-reported utilization of colonoscopy and fecal occult blood test in Europe: analysis of the European Health Interview Surveys 2013–2015 and 2018–2020

**DOI:** 10.1007/s10654-025-01247-4

**Published:** 2025-06-17

**Authors:** Idris Ola, Rafael Cardoso, Michael Hoffmeister, Hermann Brenner

**Affiliations:** 1 Division of Clinical Epidemiology and Aging Research, German Cancer Research Center (DKFZ), 69120 Heidelberg, Germany; 2 Medical Faculty Heidelberg, University of Heidelberg, 69120 Heidelberg, Germany; 3 German Cancer Consortium (DKTK), German Cancer Research Center (DKFZ), 69120 Heidelberg, Germany

**Keywords:** Colorectal cancer, Cancer screening, Colonoscopy, Fecal occult blood test, Cancer prevention and control, Utilization trends

## Abstract

Despite increasing implementation of colorectal cancer (CRC) screening programs in Europe, utilization of screening tests has varied significantly over the years. We examined recent trends in the utilization of colonoscopy and fecal occult blood test (FOBT), the primary tests recommended for CRC screening, across European countries with various screening programs. Population-based data from the second and third waves of the European Health Interview Survey (EHIS) were analyzed to determine changes in utilization of fecal occult blood test within the preceding 2 years or colonoscopy within the preceding 10 years among people aged 50–74 years between 2013–2015 and 2018–2020. Absolute percentage changes (APC) in screening test use were calculated in each country and subgroup meta-analyses were conducted using random effects models to estimate the pooled APCs and their 95% confidence intervals across different categories of screening offers. A total of 234,251 respondents across 28 European countries were included in the analysis. The increase in use of either test was highest among countries which fully rolled out nationwide organized screening programs with fecal tests between 2013–2015 and 2018–2020 (increases ranging from 19.1% units in Belgium to 46.3% units in The Netherlands) and was lowest among countries with opportunistic offering of fecal test, colonoscopy or flexible sigmoidoscopy (from − 3% units in Germany to + 12.2% units in Slovakia). Changes in screening programs were strongly associated with higher rates of utilization of colonoscopy and fecal occult blood test across all screening offers. Our findings highlight that well-organized and dynamic population screening strategies can rapidly and sustainably increase utilization of CRC screening tests in Europe.

**Supplementary Information:**

The online version contains supplementary material available at 10.1007/s10654-025-01247-4.

## Introduction

Colorectal cancer (CRC) is the second most common cause of cancer-related death and accounts for approximately one-tenth of cancer-related mortality worldwide [1], prompting a strategic focus on effective prevention and early detection efforts. In the past 3–4 decades, CRC screening has gained increasing attention for its proven ability to detect the disease at an early, treatable stage and its preventive capacity through the identification and removal of precancerous polyps [2]. As such, various fecal testing and endoscopic screening strategies have been developed to achieve mortality reduction from CRC [2, 3]. 

In Europe, the delivery strategy and utilization of CRC screening tests have witnessed dynamic changes since the adoption of the European Commission’s recommendation on CRC screening in 2003 [4]. Many European countries are increasingly embracing organized national screening programs targeting clearly defined eligible age groups based on evidence-based guidelines to optimize CRC prevention and early detection [5, 6]. However, the success of these programs strongly relies on achieving robust utilization rates, which are often influenced by socio-demographic factors, health status, and healthcare accessibility, including the types of population CRC screening offers [5, 7, 8]. 

Given the diverse and frequently changing influence of the aforementioned factors, earlier Europe-wide studies have shown wide variations in utilization estimates, with very few countries attaining the EU target of at least 65% utilization of CRC screening tests [5, 6, 8, 9]. Due to large variations in population-wide screening programs and coverage among European countries, research into the recent trends in CRC screening test utilization in Europe is essential for understanding the effectiveness of different screening approaches, identifying disparities in access and participation, and informing policy decisions to sustain enhanced screening utilization.

This paper investigates changes in utilization of fecal occult blood test (FOBT) and colonoscopy in European countries between 2013–2015 and 2018–2020, examining variations in the adoption of various population screening programs and highlighting the rates of utilization of these tests based on screening program dynamics and type of population screening offer.

## Materials and methods

### Study design and population

This analysis used population-based data from the second and third waves of the European Health Interview Survey (EHIS). EHIS is a survey of non-institutionalized individuals 15 years and older living in the EU and other participating countries [10]. Through complex population sampling and data collection methods, participants in each country provided information on four main modules: sociodemographic, health care utilization, healthcare determinants, and health status, using a uniform and standardized questionnaire, survey guidelines, and translation recommendations to achieve comparability and harmonization across countries [10]. EHIS waves 2 and 3 were carried out in 2013–2015 and 2018–2020, respectively, in all EU member countries and Iceland, Norway, Albania, Turkey, and Serbia (only EHIS-3) [10]. 

### Data collection

Data was collected through interviews and pre-tested questionnaires that were either self- or interviewer-administered onsite or online, depending on the strategies adopted by each country. The response rate averaged 60% in EHIS-2 (ranging from 26% in Luxembourg to 93% in Cyprus) and EHIS-3 (ranging from about 22% in Germany to 88% in Romania) [11, 12]. Survey weights were assigned to each respondent to account for non-response bias, the complex survey designs, and variations in the demographic distribution of the participating countries.

### Inclusion/Exclusion criteria

According to the EU Council Recommendation [4], men and women aged 50–74 years are considered eligible for CRC screening and were included in this study. Data generated through proxy interviews, both for the entire survey and CRC-screening-specific questions, were excluded to minimize reporting errors.

Since the United Kingdom (UK) did not participate in EHIS-3 and Serbia did not participate in EHIS-2, data from these countries was excluded from the analysis [11, 12]. Although France participated in the two EHIS waves, it was removed from the analysis due to inaccessibility of its EHIS-3 microdata.

### Sample size

**Fig. 1:**
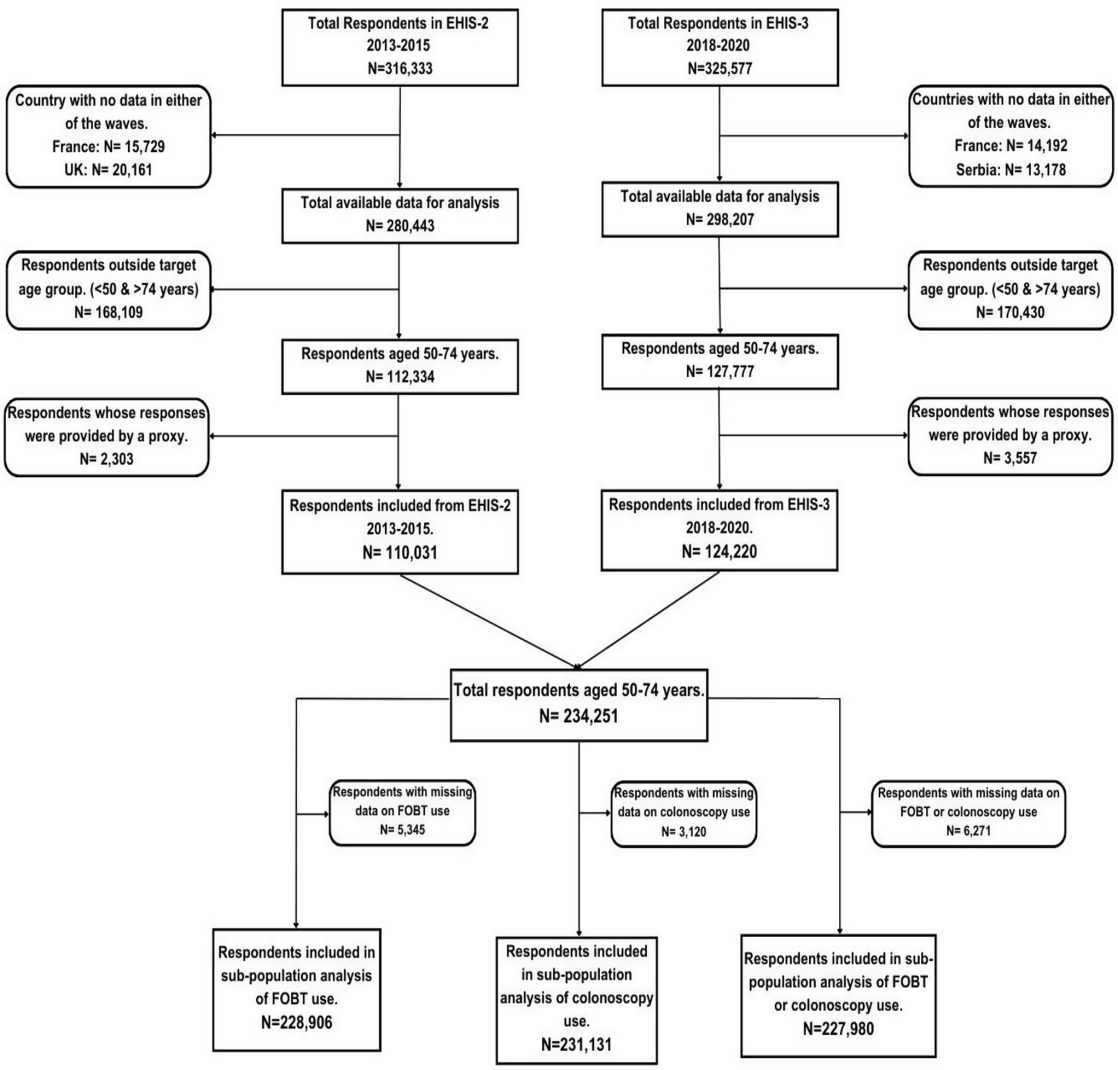
Study flowchart showing the respondents included in the final analyses. FOBT, fecal occult blood test

A total of 316,333 respondents, representing a target population of 424,755,248 people, participated in EHIS-2, while 325,577 respondents, representing 384,098,874 people, participated in EHIS-3 [11, 12]. Data from 110,031 respondents were included in the final analysis of EHIS-2 after excluding respondents from France and the UK (35,890), those outside the 50–74 age range (168,109), and data generated through proxy interviews (2,303). In EHIS-3, a total of 124,220 respondents were included in the analysis after excluding data from France and Serbia (27,370), those outside the target age group (170,430), and data provided through proxy interviews (3,557) (Fig. 1).

After excluding missing data, a subtotal of 228,906, 231,131, and 227,980 respondents were included in the final analysis of the utilization of fecal occult blood test, colonoscopy, and either test, respectively (Fig. 1).

### Measures

Outcome variables of interest are self-reported colonoscopy use within the last 10 years, fecal occult blood test (FOBT) (comprising fecal immunochemical test [FIT] or guaiac-based fecal occult blood test [gFOBT]) use within the last two years, and use of any of the two tests within the stated period prior to the interview.

Information obtained from the sociodemographic module (age, sex, education, marital status, household size, income, citizenship, employment, and location of residence) was used to describe the study population. Data collection years for EHIS-2 and EHIS-3 were used as reference periods to determine changes and trends in CRC screening test utilization.

#### Classification of countries by type of CRC screening offer and implementation status

The classification of countries by type of CRC screening offer has been described in our previous studies [5, 8]. Essentially, a comprehensive search of the CRC screening programs in all the EHIS participating countries was carried out, and their characteristics were qualitatively summarized to categorize them for the analysis of test use as shown in Supplementary Table S1. Countries were further sub-classified into two groups based on changes in their programs between EHIS-2 and EHIS-3 [5, 8]. (Table 1)

**Table 1:** Classification of countries according to CRC screening offer in years 2013–2015 and 2018–2020

Screening Offer		Offer in 2018–2020					
		None	Small scale organized program with fecal tests	Opportunistic gFOBT/FIT only	Opportunistic gFOBT/FIT or colonoscopy	Organized gFOBT/FIT (partial)	Organized gFOBT/FIT (full)
**Offer in** **2013–2015**	**None**	Bulgaria (50–74)	Estonia (50–74)	NA	Luxembourg [**50–54]**	Hungary (50–69**)/[50–74]**	Slovenia (70–74)
		Cyprus (50–74)	Hungary (70–74)		Luxembourg [**55–74]**	Ireland (55–59, 70–74)	
		Norway (50–74)	-		-	Malta (65–74)	
		Poland (50–74)				-	
		Romania (50–74)					
	**Small scale organized program with fecal tests**	NA	NA	NA	NA	NA	NA
	**Opportunistic gFOBT/FIT only**	NA	NA	Latvia (50–74)	NA	NA	NA
	**Opportunistic gFOBT/FIT or colonoscopy**	NA	Luxembourg (50–74)	NA	Austria (50–74)	Czechia (50–74)	NA
			-		Germany (50–74)	Portugal (50–74)	
					Greece (50–74)	-	
					Iceland (50–74)		
					Slovakia (50–74)		
	**Organized gFOBT/FIT only (partial)**	Finland (60–69)/[**60–74]**	NA	NA	NA	Ireland (60–69)/**[60–74]**	Belgium (50–74)
						Italy (50–69)/**[50–74]**	Denmark (50–74)
						Malta (55–64)/**[55–69]**	Lithuania (50–74)
						Spain (50–69)/**[50–74]**	Netherlands (55–74)
						Sweden (60–69)/**[60–74]**	-
	**Organized gFOBT/FIT only (full)**	NA		NA	NA	NA	Croatia (50–74)
							Slovenia (50–69)/**[50–74]**

Age groups in bold font and square bracket indicate the age group included in analysis of colonoscopy use and use of either test. gFOBT, guaiac-based fecal occult blood test; FIT, fecal immunochemical test; NA, not applicable

1. Sub-group I: Countries and age groups with stable programs across the two EHIS waves.
2. Sub-group II: Countries and age groups that transitioned from one program to another between EHIS-2 and EHIS-3.

To analyze CRC screening test utilization, within each sub-group, countries were categorized as follows:

A. Countries with organized, fully rolled-out programs using fecal tests.
B. Countries with organized fecal test-based programs, partially rolled-out, or with regional coverage only.
C. Countries with opportunistic programs using fecal tests, colonoscopy, or flexible sigmoidoscopy.
D. Countries with no program or only a small-scale organized pilot program using fecal tests.

### Analytical approach

Descriptive analyses were used to characterize the study participants. The weighted proportion of screening utilization in each wave was graphically presented for each country in the two sub-groups of countries that did and did not implement changes in screening offers (as categorized above).

The absolute percentage change (APC) (and the standard errors) in use of FOBT, colonoscopy, and either test was calculated from the weighted proportions for 2013–2015 and 2018–2020 in each country. A subgroup meta-analysis was conducted to calculate the APC and their 95% confidence intervals across different categories of screening offers using the meta function in R [13]. For each category, we derived pooled estimates and an overall estimate across all categories using a random-effects model. Forest plots were generated to visualize these pooled estimates and assess the heterogeneity within and between subgroups, providing a comprehensive overview of the changes in screening utilization over the study period.

All analyses were conducted using R Statistical Software (Version 4.3.2) [14]. All tests were 2-tailed, and an alpha error < 0·05 was taken as statistically significant.

### Ethical consideration

The legal authorization for EHIS surveys was provided by “Regulation (EC) No 1338/2008 of the European Parliament and of the Council of December 16, 2008 on Community Statistics on Public Health and Health and Safety at Work (Text with EEA Relevance)” [15]. According to Commission Regulation (EU) Nos. 141/2013 and 2018/255, the data collected by each country and the methods of data collection for both EHIS waves are described [11, 12]. Ethical clearances were also obtained at the national level by the respective institutions that conducted the survey in each participating country. Access to the EHIS microdata for analysis of each wave was granted by Eurostat with proposal number RPP 294/2022-EHIS.

## Results

Main characteristics of the study population are presented in Table 2. There was fairly equal age group and sex representation of participants, with females constituting 52.4% in both waves and a gradual decline of proportions of participants in five-year age groups from approximately 24% in 50–54 to 15% in 70–74 age groups in both waves. Approximately two thirds of participants were married, and approximately two thirds lived in households with one or two people in both waves. Characteristics for educational and income levels, employment status, residence, and citizenship were also similar between the waves.

**Table 2:** Sociodemographic characteristics of respondents in EHIS waves 2 and 3

Characteristics		N [Weighted % (95% CI)]	
		EHIS-2	EHIS-3
		2013–2015 (*N* = 110,031)	2018–2020 (*N* = 124,220)
Age group (Year)	50–54	24,245 [**24.3** (23.9, 24.6)]	24,922 [**23.9** (23.5, 24.3)]
	55–59	23,800 [**22.2** (21.9, 22.5)]	25,569 [**21.7** (21.3, 22.0)]
	60–64	23,098 [**20.8** (20.2, 21.1)]	26,231 [**21.1** (20.7, 21.4)]
	65–69	21,513 [**17.9** (17.6, 18.2)]	25,504 [**18.2** (17.9, 18.5)]
	70–74	17,375 [**14.8** (14.6, 15.1)]	21,994 [**15.2** (14.9, 15.5)]
Sex	Male	50,067 [**47.6** (47.2, 48.0)]	56,974 [**47.6** (47.2, 48.1)]
	Female	59,964 [**52.4** (52.0, 52.8)]	67,246 [**52.4** (51.9, 52.8)]
Marital status	Married/registered partners	74,630 [**71.4** (71.1, 71.8)]	81,949 [**65.7** (65.2, 66.1)]
	Never Married	10,104 [**8.3** (8.1, 8.6)]	13,882 [**11.9** (11.5, 12.2)]
	Widowed or divorced	25,019 [**20.2** (19.9, 20.6)	28,107 [**22.5** (22.1, 22.9)]
Educational level ^a^	Tertiary education	23,187 [**21.7** (21.3, 22.0)]	32,107 [**22.4** (22.1, 22.7)]
	Upper secondary	46,776 [**43.3** (42.9, 43.7)]	53,109 [**47.4** (47.0, 47.9)]
	No education or less than upper secondary	39,497 [**35.0** (34.7, 35.4)]	38,405 [**30.2** (29.8, 30.6)]
Residence	City	36,056 [**35.2** (34.8, 35.6)]	41,647 [**36.5** (36.1, 36.9)]
	Town or suburb	32,813 [**33.7** (33.3, 34.1)]	41,449 [**36.4** (35.9, 36.8)]
	Rural area	41,139 [**31.1** (30.7, 31.5)]	40,704 [**27.2** (26.8, 27.5)]
Employment status ^b^	Employed	42,980 [**42.8** (42.4, 43.2)]	52,149 [**45.8** (45.4, 46.3)]
	Unemployed and others	20,636 [**19.0** (18.7, 19.3)]	20,421 [**17.5** (17.1, 17.8)]
	Retired	45,744 [**38.3** (37.9, 38.6)]	51,032 [**36.7** (36.3, 37.2)]
Income level ^c^	Quintiles 4 and 5	43,203 [**43.5** (43.1, 43.9)]	50,484 [**44.5** (44.1, 45.0)]
	Quintile 3	21,164 [**20.1** (19.8, 20.4)]	24,112 [**19.7** (19.4, 20.1)]
	Quintiles 1 and 2	38,736 [**36.4** (36.0, 36.8)]	42,611 [**35.8** (35.3, 36.2)]
Citizenship ^d^	Native-born	106,705 [**97.4** (97.2, 97.5)]	116,664 [**96.6** (96.4, 96.8)]
	Non-natives	3,041 [**2.6** (2.5, 2.8)]	4,385 [**3.4** (3.2, 3.6)]
Household size	2 people or less	76,095 [**64.0** (63.6, 64.4)]	87,391 [**65.7** (65.3, 66.2)]
	3 people or more	33,802 [**36.0** (35.6, 36.4)]	36,572 [**34.3** (33.8, 34.7)]

(a) Educational level was categorized based on ISCED classification. Tertiary level includes short-cycle tertiary education, bachelor’s or equivalent level, master’s or equivalent level, and doctoral or equivalent level. Upper secondary category also includes post-secondary non-tertiary education and No education or less than upper secondary level includes: No formal education or below ISCED 1, primary, and lower secondary education. (b) Unemployed and others include those that are unemployed, student, pupil, engaged in domestic tasks, unable to work due to longstanding health problems, on compulsory military or civilian service, and others not classified. (c) Based on self-reported total equivalized disposable household income. (d) Non-native category include respondents born in another European country and in a non-European country

### Changes in CRC screening offers in the EU countries, Iceland, and Norway

Most countries maintained stable programs across the two periods, and a few have transitioned in program type, coverage, or target age groups. Programs in five countries (Belgium, Denmark, Lithuania, the Netherlands, and Slovenia (70–74) became fully rolled-out between the two periods, whereas five countries, overall or within specific age groups (Portugal, Hungary, Malta (65–74), Czechia, and Ireland (55–59, 70–74)) transitioned to an organized screening program with partial or regional coverage. While all countries running opportunistic screening with fecal tests, colonoscopy, or sigmoidoscopy remained stable in the reference period, only Luxembourg transitioned by target age group in this category. Three countries (Hungary (70–74), Estonia, and Luxembourg) transitioned to the category of countries with small-scale organized pilots with fecal tests, while Finland transitioned to no program from its initial partially rolled-out organized program with fecal tests (Table 1).

### Changes in CRC screening utilization rates by type of CRC screening offer

**Fig. 2:**
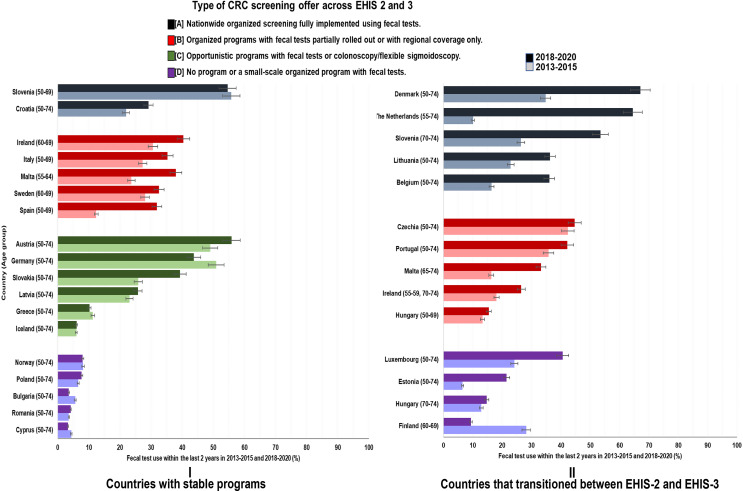
Changes in utilization of fecal tests (with 95% CI) by type of CRC screening offer

**Fig. 3:**
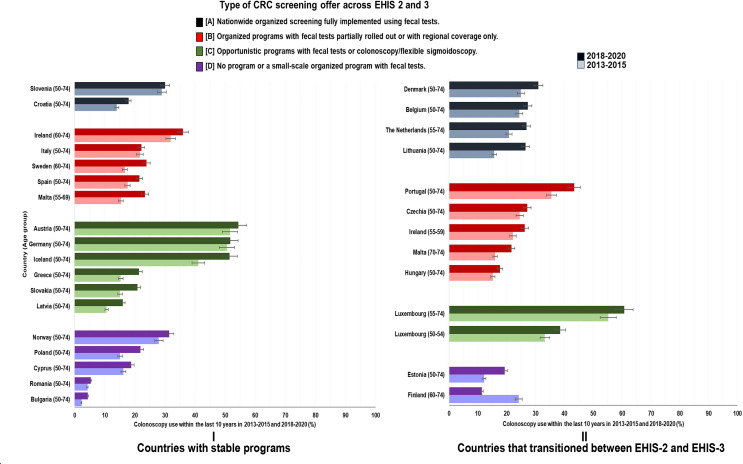
Changes in utilization of colonoscopy (with 95% CI) test by type of CRC screening offer

**Fig. 4:**
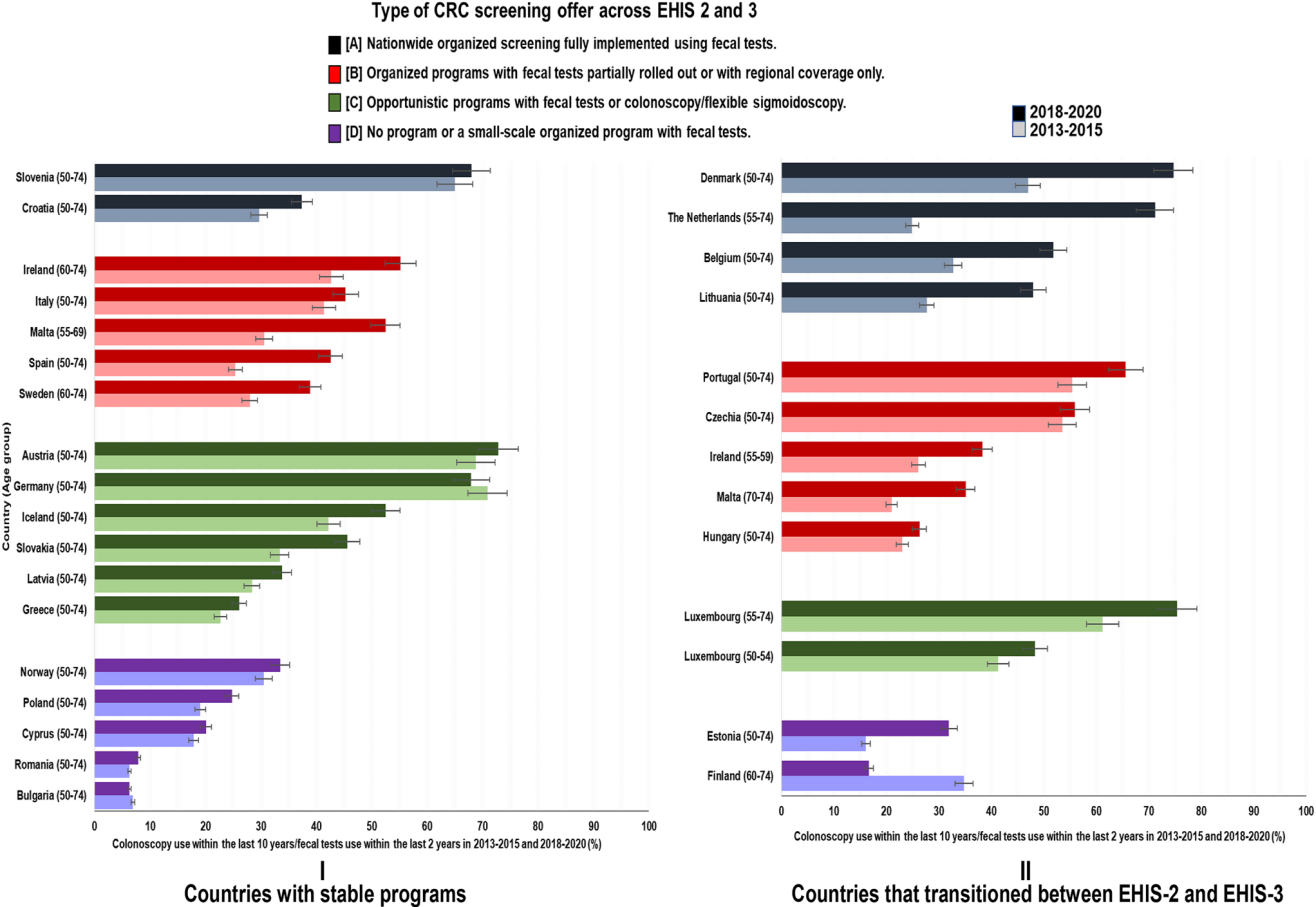
Changes in utilization of either fecal test or colonoscopy (with 95% CI) by type of CRC screening offer

Across all screening offers, utilization rates generally increased in varying proportions in 2018–2020 compared to 2013–2015. However, the strongest increases and the highest rates in 2018–2020 were seen among countries that transitioned between the two periods (Figs. 2, 3 and 4).

The most impressive increases in FOBT use were seen in countries transitioning to fully rolled-out organized programs with fecal tests, such as the Netherlands (+ 54.4% units, from 10.1% (95% CI: 8.8–11.3) to 64.5% (95% CI: 62.6–66.5)), Denmark (+ 32.3% units, from 34.8% (95% CI: 32.9–36.6) to 67.1% (95% CI: 65.4–68.8)) and Belgium (+ 19.7% units, from 16.4% (95% CI: 14.4–18.5) to 36.1% (95% CI: 34.0-38.3)) (Fig. 2, Supplementary Table S2). In Slovenia, where the screening program was extended to age group 70–74, a strong increase of FOBT use was selectively seen in this age group (+ 27.1% units, from 26.4% (95% CI: 21.7–31.1) to 53.5% (95% CI: 49.4–57.7)), whereas utilization rates remained essentially stable at approximately 55% at ages 50–69. Countries with partially rolled out organized screening programs mostly experienced modest increases in use of FOBT. Divergent trends were observed in countries offering opportunistic screening programs in both periods. Whereas FOBT use increased in Austria (+ 6.9% units, from 49.0% (95% CI: 47.5–50.5) to 55.9% (95% CI: 54.4–57.4)) and Slovakia (+ 13.4% units, from 25.9% (95% CI: 24.0-27.9) to 39.3% (95% CI: 37.4–41.3)), a decline was observed in Germany (− 7.1% units, from 50.9% (95% CI: 49.8–52.0) to 43.8% (95% CI: 42.4–45.2)). Countries transitioning to no program also demonstrated notable increases in use of FOBT except Finland, where FOBT use within the previous 2 years dropped by 19% units, from 28.2% (95% CI: 27.4–29.0) to 9.4% (95% CI: 7.7–11.1).

Use of colonoscopy within the past 10 years strongly varied between countries but increased in all countries except Finland, reaching levels > 50% in Luxembourg, Austria, Germany and Iceland in 2018–2020, whereas utilization rates remained close to 5% in Romania and Bulgaria and fell back to little over 10% in Finland (Fig. 3, Supplementary Table S3).

As shown in Fig. 4 and Supplementary Table S4, the use of either screening test increased in all countries between 2013–2015 and 2018–2020 except Finland and Bulgaria, with increases generally being most pronounced in countries with fully or partially rolled out organized screening programs.

Changes in test utilization stratified by sex and age groups showed great variability, but with an overall relatively larger rate increase in men and among the age group 60–74 years. (Supplementary Figures S1-S8)

### Pooled estimates of absolute changes in percentage use of CRC screening tests by type of screening offer between EHIS-2 and EHIS-3

The overall random effects model for FOBT use indicated a substantially higher and statistically significant increase in the absolute rate of FOBT use in transitioned countries (+ 14.1% units, 95% CI: 5.1–23.1) compared to stable countries, which showed a modest but statistically insignificant increase of 4.1% units (95% CI: 0.9–7.2) (Supplementary Figure S9). The pooled estimates for colonoscopy use showed equal rate of increase in both stable countries (+ 4.0% units; 95% CI: 2.8–5.2) and transitioning countries (+ 4.1% units; 95% CI: 0.9–7.3), although the increase was statistically insignificant in the latter (Supplementary Figure S10).

Overall, transitioning countries demonstrated a two-fold higher absolute increase in utilization of either test (+ 13.5% units; 95% CI: 5.3–21.6) compared to stable countries (+ 6.6% units; 95% CI: 3.7–9.5) (Supplementary Figure S11).

## Discussion

Our study investigated changes in the utilization rates of fecal occult blood test and colonoscopy among average-risk individuals aged 50–74, as reported in the European Health Interview Surveys in 2013–2015 and 2018–2020 across 28 European countries, with varying types of CRC screening programs. Overall, most countries demonstrated increases in utilization of these tests in 2018–2020 compared to the levels achieved in 2013–2015. However, countries that transitioned to or expanded organized screening programs exhibited more significant progress. Despite these improvements, there remains much room for enhanced offers and more effective use of CRC screening across European countries.

In countries with fully rolled-out well-organized programs with fecal tests, transitioning countries like the Netherlands and Denmark demonstrated particularly strong increases in screening utilization. In Denmark, the implementation of the first screening round of the Danish National Colorectal Cancer Screening Program in 2014–2017 resulted in a high uptake, with an average of 58.4% of invited men and 66.6% of women undergoing screening [16]. The rapid attainment of national coverage in this program culminated in overall utilization rates surpassing the European target by over 2% units as of 2020 [8], mirroring a similar trend observed in the Netherlands. In Lithuania and Belgium, the transition from regional to national coverage of screening programs, along with the expansion of screening programs into the large Flemish region of Belgium, likely contributed to the rapid increase in participation. In Slovenia and Croatia on the other hand, where stable, long-running programs have been in place, observed rates were much lower.

While countries with partially rolled-out programs with fecal tests generally exhibited lower utilization rates compared to those with full national coverage, utilization rates also generally increased in these countries. There were, however, some notable differences between the countries which may be explained by the specifics of the various programs. For example, while Portugal and Czechia transitioned from predominantly opportunistic programs, Hungary, Ireland (age groups 50–59), and Malta (age groups 70–74) transitioned from no screening program to partially implemented organized programs. Furthermore, factors such as level of community awareness, population coverage, resource allocation and management, as well as technical proficiency, may also play a major role. For instance, in Czechia, the response to repeat screening invitations was reportedly poor, even with a slight decline noted around 2016 [17]. Similarly, public awareness and information dissemination in Hungary seem to have been inadequate, with a survey indicating that up to 27% of respondents in 2018 were unaware of any CRC screening modalities [18]. 

Among countries offering opportunistic programs, both countries with stable and transitioning programs mostly showed significant percentage increases in CRC tests utilization. A notable exception was Germany, where use of FOBT was lower in 2018–2020 than in 2013–2015. This observation aligns with a previous report indicating a progressive decline in fecal test utilization in Germany [19]. This observation is a setback as first very preliminary steps towards an organized screening program were taken in 2019 in that a single information letter on screening was sent, for the first time, to people aged 50, 55, 60 and 65. Our data suggest that this type of intervention may not have had any beneficial effect on use of CRC screening offers, and more comprehensive efforts towards an organized screening program are needed to achieve such effects.

The observed changes in countries with no screening programs or small-scale pilot programs at the time of the surveys typically exhibited a predictable pattern of inconsistent test utilization. It is plausible that these patterns primarily reflect usage for symptomatic purposes [5], particularly considering the prominence of colonoscopy-driven estimates in this group in our study. However, Estonia, a notable outlier in this group, demonstrated rapid utilization of FOBT. This can be attributed to the early impacts of a small-scale, organized pilot screening program that commenced in 2016 [20]. In Finland, another significant outlier in this group, the striking decline in CRC screening test utilization between EHIS-2 and EHIS-3 reflects utilization during the peak of an earlier program and the early phase of a successor program. From 2004 to 2014, a gFOBT-based population screening program was gradually implemented nationwide on an experimental basis, with individuals randomized into screening and non-screening arms based on their region, sex, and year of birth [21, 22]. Inconsistent findings during evaluation of that program led to a temporary closure of the program in 2015 before its re-launch in the following year. Our data suggests that utilization rates in 2018–2020 had not yet recovered from this temporary closure of the program which eventually fully took off in 2019 during the period of EHIS-3 data collection [22]. 

Overall, countries with organized, fully rolled-out programs demonstrated the highest percentage increase in CRC screening test utilization. This emphasizes the crucial role of wide-reaching, organized population-based CRC screening programs in driving screening test utilization, echoing findings from Denmark, where an active public health program emerged as the primary driver for individuals’ participation in CRC screening [23]. Additionally, variations in screening test utilization across countries may be influenced by differences in program implementation strategies, particularly whether FOBT kits are directly mailed to eligible individuals or require active requests. Evidence suggests that direct mailing and reminder systems, such as follow-up letters or text messages, significantly enhance participation rates within organized screening programs [24, 25]. 

When compared with other trend analyses of CRC screening test utilization, our findings, although comparing different types of population screening offers, are largely consistent with reports from other countries where screening facilities are readily available [9, 26–28]. In the United States, where CRC screening is primarily opportunistic, utilization increased by 10% from 2008 to 2015 (from 51.6 to 61.3%) [28]. Furthermore, there was a subsequent swift increase of 4%, reaching 65% in 2018. This increase was largely attributed to a resurgence in fecal testing, particularly with the adoption of newer methods such as the multitarget fecal DNA test since 2015 [6, 29]. 

In South Korea, utilization of CRC screening tests experienced a rapid surge, increasing by 35% points from 25.0 to 60.1% between 2005 and 2014. However, this upward trend slowed significantly thereafter, with just 4% units increase (to 64.4%) recorded between 2014 and 2020 [30], suggesting potential ceiling effects once most of the population open to preventive actions has been reached. It will remain to be seen to what extent well-organized screening programs or special efforts to target the hard-to-reach population groups which may also be at higher risk of CRC due to less healthy lifestyles, may be able to surpass such use rates in the future.

### Strengths and limitations

The findings in this study relied on large, nationally representative, population-based data, giving appreciable statistical power and allowing for comparison of trends among many countries with highly divergent screening program implementation. The report of estimates and effect sizes by the type of CRC screening offer in each country provided a unique opportunity to evaluate how screening programs and changes within programs may affect utilization of the tests, giving further opportunities and insights for country- and continental-level improvement of the programs.

Since the data was based on self-reported information, the risk of reporting and recall biases could not be ruled out. It should also be noted that there was no sufficient clarification between screening and diagnostic tests in the CRC-screening test-specific questions in the EHIS surveys used in this study, therefore, the screening utilization rates reported in this study, especially in countries with limited screening programs, may have been overestimated through reports of diagnostic testing. This should not have been a major issue for fecal tests, which are mostly recommended in Europe as non-invasive screening tools (but may also play a role in guiding diagnostic assessments in some clinical settings, particularly in symptomatic individuals). By contrast, some non-negligible proportion of colonoscopies most likely was performed to follow up symptoms rather than as primary screening exam. Also, since we looked at countries with recent changes or commencement of their screening programs, the 10-year interval for analysis of colonoscopy use might have introduced some imprecision in utilization rates in countries which offered screening since less than 10 years.

Although we have analyzed the utilization of these tests within the context of existing population-based screening program structures, it should also be emphasized that test utilization patterns may also be influenced by factors beyond the type of screening programs, such as improved healthcare access, evolving clinical guidelines, and demographic shifts, among others.

## Conclusions

Our analyses reveal highly divergent dynamics in achieving target goals for CRC screening in European countries which appear to be closely linked to organizational features of the screening programs. Our results clearly demonstrate the potential, merits and necessity of well-organized screening programs to achieve CRC screening targets. Achieving such targets will be of paramount importance to cope with the increasing CRC burden in the decades to come that is otherwise to be expected due to the demographic development and increasing CRC incidence in younger birth cohorts currently observed in many countries within and beyond Europe [31, 32]. 

To improve the utility of future EHIS surveys in monitoring CRC screening trends, we recommend that subsequent waves include clearer distinctions in the preventive healthcare services module, differentiating between tests conducted for screening purposes and those performed for diagnostic or symptomatic evaluation. This refinement would enhance the accuracy of screening utilization estimates and facilitate more targeted public health interventions.

## Electronic supplementary material

Below is the link to the electronic supplementary material.

Supplementary Material 1

## Data Availability

Access to study data (EHIS waves 2 and 3) was granted by Eurostat. Information on how to access the data can be found at https://ec.europa.eu/eurostat/web/microdata/european-health-interview-survey.

## Declarations

### Disclaimer

The study’s results and conclusions are authors’ responsibility and do not represent the views or opinions of Eurostat, the European Commission or any national statistical authority of the included countries.
