# Supplementary material for: Recent trends in self-reported utilization of colonoscopy and fecal occult blood test in Europe: analysis of the European Health Interview Surveys 2013–2015 and 2018–2020
Source: Eur J Epidemiol. 2025 Jun 17;40(7):767–78. doi: 10.1007/s10654-025-01247-4 (PMC12304025; doi:10.1007/s10654-025-01247-4)
Supplement: Supplementary file 1 — Supplementary Material 1 [file 10654_2025_1247_MOESM1_ESM.docx]

# **Supplementary document**

# **Recent trends in self-reported utilization of colonoscopy and fecal occult blood test in Europe: Analysis of the European Health Interview Surveys 2013-2015 and 2018-2020.**

**Idris Ola,^1,2^**^,^ **Rafael Cardoso,^1^ Michael Hoffmeister,^1^ Hermann Brenner,^1,3*^**

^1^Division of Clinical Epidemiology and Aging Research, German Cancer Research Center (DKFZ), 69120 Heidelberg, Germany.

^2^Medical Faculty Heidelberg, University of Heidelberg, 69120 Heidelberg, Germany

^3^German Cancer Consortium (DKTK), German Cancer Research Center (DKFZ), 69120 Heidelberg, Germany

**^*^**Corresponding author: Prof. Dr. med. Hermann Brenner, Division of Clinical Epidemiology and Aging Research, German Cancer Research Center (DKFZ), 69120 Heidelberg, Germany.

Email: [h.brenner@dkfz-heidelberg.de](mailto:h.brenner@dkfz-heidelberg.de); Tel.: +49-6221-42-1300

**Table of contents**

**Page**

Table of contents 2

Table S1. Classification of EHIS-participating countries by type of CRC screening programs. 3-5

Table S2. Changes in utilization of FOBT by type of CRC screening offer in 2013-2015 and 2018-2020. 6

Table S3. Changes in utilization of colonoscopy by type of CRC screening offer in 2013-2015 and 2018-2020. 7

Table S4. Changes in utilization of either test by type of CRC screening offer in 2013-2015 and 2018-2020. 8

Figure S1. Utilization rate of FOBT, colonoscopy, and either test by sex for countries in category A 9

Figure S2. Utilization rate of FOBT, colonoscopy, and either test by sex for countries in category B 10

Figure S3. Utilization rate of FOBT, colonoscopy, and either test by sex for countries in category C 11

Figure S4. Utilization rate of FOBT, colonoscopy, and either test by sex for countries in category D 12

Figure S5. Utilization rate of FOBT, colonoscopy, and either test by age for countries in category A 13

Figure S6. Utilization rate of FOBT, colonoscopy, and either test by age for countries in category B 14

Figure S7. Utilization rate of FOBT, colonoscopy, and either test by age for countries in category C 15

Figure S8. Utilization rate of FOBT, colonoscopy, and either test by age for countries in category D 16

Figure S9. Forest plot shows pooled absolute percentage change in use of gFOBT/FIT for each category between stable and transitioned countries. 17

Figure S10. Forest plot showing pooled absolute percentage change in use of colonoscopy for each category between stable and transitioned countries. 18

Figure S11. Forest plot showing pooled absolute percentage change in use of either test for each category between stable and transitioned countries. 19

Supplementary reference list (for Table S1) 20-22

**Table S1.** Classification of EHIS-participating countries by type of CRC screening programs.^8^

| **Country** | **Structure of screening program** | **Year of program initiation** | **Age group** | **Type of screening test** | **Screening interval** | **References^##^** | **Category according to screening offer**** |
| --- | --- | --- | --- | --- | --- | --- | --- |
| **Belgium** (Flemish region) | Organized | 2013-2018 | 56-74 | FIT | 2 yrs | ^34, 35^ | A |
|  |  | 2018-2020 | 53-74 |  |  |  |  |
|  |  | 2020 | 50-74 |  |  |  |  |
| **Belgium** (Wallonia/Brussels) |  | 2009-2016 | 50-74 | gFOBT |  |  |  |
|  |  | 2016 |  | FIT |  |  |  |
| **Croatia** | Organized | 2007 | 50-74 | gFOBT | 2 yrs | ^36-38^ |  |
| **Denmark** | Organized | 2014 | 50-74 | FIT | 2 yrs | ^39^ |  |
| **Lithuania** (Vilnius and Kaunas) | Organized | 2009-2014 (pilot) | 50-74 | FIT | 2 yrs | ^40, 41^ |  |
| **Lithuania** (All regions) |  | 2014 |  |  |  |  |  |
| **The Netherlands** | Organized | 2014 | 55-75 | FIT | 2 yrs | ^42^ |  |
| **Slovenia** | Organized (pilot) | 2008-2009 | 50-69 | FIT | 2 yrs | ^43, 44^ |  |
|  | Organized | 2009-2015 | 50-74 |  |  |  |  |
|  |  | 2015 |  |  |  |  |  |
|  | | | | | | | |
| **Czechia** | Organized (pilot) | 1979-1998 | 45-60 | gFOBT | 2 yrs | ^5, 45-47^ | B |
|  | Opportunistic | 2000-2009 | 50-54 |  |  |  |  |
|  | Opportunistic | 2009 | 50-54 | FIT/Colo | 1 yr |  |  |
|  | Opportunistic | 2009 | 55+ | FIT/Colo | 2 yrs/10 yrs |  |  |
|  | Organized | 2014 | 50+ | FIT | 2 yrs |  |  |
| **Hungary (**Csongrád county) | Organized | 2013-2015 | 50-70 | FIT | 2 yrs | ^48, 49^ |  |
| **Hungary^(a)^** | Organized | 2018 |  |  |  |  |  |
| **Ireland** | Organized | 2012 | 60-69  (Later 55-74) | FIT | 2 yrs | ^36, 50, 51^ |  |
| **Italy** | Organized | 1982-1996 | 50-69 | gFOBT | 2 yrs | ^52-54^ |  |
|  | Organized | 1996 | 50-70/74 | FIT |  |  |  |
| **Italy** (Piedmont and Veneto regions) | Organized | 2003/2004 | 58-60 | FS | Once only |  |  |
| **Malta** | Organized | 2012 | 55-66 | FIT | 2 yrs | ^38, 55^ |  |
|  |  | NR | 55-74 |  | 2 yrs |  |  |
| **Portugal** | Opportunistic | NR | 50-74 | FIT/Colo | 1 yr/10 yrs | ^5, 56-58^ |  |
| **Portugal** (Alentejo and Central regions) | Organized | 2009-2018 | 50-70 | gFOBT | 2 yrs |  |  |
|  |  | 2018 |  | FIT |  |  |  |
| **Portugal**  (Northern region) | Organized (pilot) | 2016-2018 | 50-74 | FIT |  |  |  |
|  | Organized | 2018 |  |  |  |  |  |
| **Spain** | Organized | 2000-2010 | 50-69 | gFOBT | 2 yrs | ^5, 36, 59-61^ |  |
|  |  | 2010 |  | FIT |  |  |  |
| **Sweden** (Regions Gotland and Stockholm) | Organized | 2008-2015 | 60-69 | gFOBT | 2 yrs | ^38, 54, 62^ |  |
|  |  | 2015 |  | FIT |  |  |  |
| **Sweden** (Region Skåne) |  | 2021 | 60-74 | FIT |  |  |  |
|  | | | | | | | |
| **Austria** (Burgenland) | Organized | 2003 | 40-80 | FIT | 1 yr | ^54, 63^ | C |
| **Austria** (Vorarlberg)^(b)^ |  | 2007 | 50+ | Colo | 10 yrs |  |  |
| **Austria** (All regions) | Opportunistic | 1980 | 40+ | gFOBT, | 1 yr |  |  |
|  |  | 2005 | 50+ | Colo | 7-10 yrs |  |  |
| **Germany** | Opportunistic | 1977-2002 | 45+ | gFOBT | 1 yr | ^20, 64, 65^ |  |
|  | Opportunistic | 2002-2017 | 50-54 | gFOBT | 1 yr |  |  |
|  |  | 2017 |  | FIT |  |  |  |
|  | Opportunistic | 2002-2017 | 55+ | gFOBT/Colo | 2 yrs/10 yrs |  |  |
|  |  | 2017 |  | FIT/Colo |  |  |  |
|  | Organized | 2019 | 50-54/55+ | FIT | 1 yr, then 2 yrly |  |  |
|  | Organized | 2019 | 50+(men); 55+(women) | Colo (up to 2 screenings) | 10 yrs |  |  |
| **Greece** | Opportunistic | NR | 50-74 | gFOBT | NR | ^36, 61^ |  |
|  |  |  |  | Colo | NR |  |  |
| **Latvia** | Opportunistic | 2005 | 50-74 | gFOBT | 1 yr | ^36, 66^ |  |
| **Slovakia** | Opportunistic | 2002-2019 | 45-75 | gFOBT/Colo | 2 yrs | ^67^ |  |
|  | Organized (pilot) | 2019-2021 | 50-75 | FIT |  |  |  |
|  | Organized | 2021 |  | FIT |  |  |  |
|  | | | | | | | |
| **Bulgaria^(c)^** | Opportunistic | 2009 | NR | gFOBT | NR | ^68^ | D |
|  | No program | 2009 |  | NA | NA |  |  |
| **Cyprus^(d)^** | No program | NA | NA | NA | NA | ^69^ |  |
| **Estonia^(e)^** | Organized (Pilot) | 2016 | 60-69 | FIT | 2 yrs | ^70, 71^ |  |
|  | Organized | 2022 |  |  |  |  |  |
| **Finland** | Organized | 2004-2016 | 60-69 | gFOBT | 2 yrs | ^23, 72^ |  |
|  | Organized (pilot) | 2019-2027 | 56-74 | FIT |  |  |  |
| **Iceland** | Opportunistic | NR | 50+ | Colo | NR | ^73^ |  |
| **Luxembourg** | Opportunistic | 2005- | 50+ | gFOBT/Colo | NA | ^5, 74^ |  |
|  | Organized (Pilot) | 2016-2019 | 55-74 | FIT/Colo | 2 yrs/10 yrs |  |  |
|  | Organized | 2021 |  | FIT | 2 yrs |  |  |
| **Norway** (Østfold, Akershus & Buskerud counties) | Organized (pilot) | 2012-2018 | 50-74 | FS/FIT | 2 yrs (FIT),  Once (FS) | ^75-78^ |  |
| **Norway** (All regions) **^(g)^** | Organized | 2022 | 55+ | FIT | 2 yrs |  |  |
| **Poland** | Opportunistic | 2000-2011 | 50-66 | Colo | 10 yrs | ^79-81^ |  |
| **Poland** (25 of 380 counties) | Organized (pilot) | 2012 | 55-64 | Colo | Once only |  |  |
| **Romania** | No program | NA | NA | NA | NA | ^82^ |  |

**^##^** Reference list for Table S1. See Supplementary reference list in this Supplementary Material. Note that the reference numbering and listing here is a continuation of the numbering from the main document.

Abbreviations: gFOBT, guaiac-based fecal occult blood test; FIT, fecal immunochemical test; FS, flexible sigmoidoscopy; Colo, colonoscopy; NA, not applicable; NR, not reported; yr(s), year(s).

******Category A, countries with organized, fully rolled-out programs using fecal tests;

Category B, countries with organized fecal test-based programs, partially rolled-out, or with regional coverage only; Category C, countries with opportunistic programs using fecal tests, colonoscopy or flexible sigmoidoscopy; Category D, countries with no program or only a small-scale organized pilot program using fecal tests.

^(a)^ In Hungary, eligible persons are invited by the National Public Health Institute based on their association with general practitioners (GPs) who are participating in the screening program. The GPs are reimbursed per screened individual.^(supp. ref. list, ref. 49)^ ^(b)^ In Austria (Vorarlberg), screening is only available for insured individuals according to the protocol.^(supp. ref. list, ref. 54) (c)^ In Bulgaria, general practitioners (GPs) were responsible for the referral of patients for gFOBT until 2009, when the opportunistic program was discontinued due to poor compliance by both the practitioners and the eligible persons.^(supp. ref. list, ref. 68)^ ^(d)^ In Cyprus, a small-scale pilot program with fecal tests was said to have been conducted in 2013; however, no further information has been provided about the continuation of any screening program since then. ^(e)^ In Estonia, an organized screening program was piloted among insured persons from 2016 until 2022, when it was expanded to all eligible individuals. ^(f)^ In Finland, following the pilot of FIT in 2019-2021 in 12 volunteer municipalities, the Government Decree on Screenings was amended to make screening available nationwide from 2022 onwards. Screening every 2 years was initially introduced among 60–68-year-olds and will be expanded to all target age groups (56-74) by 2031. (<https://cancerregistry.fi/screening/colorectal-cancer-screening/>) ^(g)^ In Norway, organized screening with FIT was piloted between 2012 and 2018 in Østfold, Akershus, and Buskerud Counties. Since May 2022, the Norwegian Cancer Registry has administered a nationwide CRC screening with FIT every 2 years from age 55. The program is expected to be replaced with a once-only screening colonoscopy after five FIT screening rounds, when colonoscopy capacity would have improved to cover the entire target population.^(supp. ref. list, ref. 75)^

**Table S2**. Changes in utilization of fecal occult blood test by type of CRC screening offer in 2013-2015 and 2018-2020.

| **Category** | **Country (Age)** | **2013-2015** | | **2018-2020** | |
| --- | --- | --- | --- | --- | --- |
|  |  | **n (test users)/ N (total respondents)** | **Weighted % (95% CI)** | **n (test users)/ N (total respondents)** | **Weighted % (95% CI)** |
| **Countries with stable programs** | | | | | |
| A | Slovenia (50-69) | 1110 / 1957 | 55.8 (53.4-58.2) | 1929 / 3433 | 54.6 (53.0-56.5) |
|  | Croatia (50-74) | 451 / 2091 | 22.0 (20.0-23.9) | 677 / 2358 | 29.2 (26.8-31.7) |
|  | | | | | |
| B | Ireland (60-69) | 489 / 1611 | 30.6 (28.4-32.9) | 526 / 1348 | 40.4 (37.2-43.5) |
|  | Italy (50-69) | 2387 / 7507 | 27.3 (26.9-27.7) | 5099 / 14,236 | 35.3 (34.4-36.2) |
|  | Malta (55-64) | 183 / 768 | 23.7 (20.7-26.8) | 298 / 781 | 38.0 (34.5-41.4) |
|  | Sweden (60-69) | 177 / 757 | 28.1 (27.4-28.9) | 479 / 1390 | 32.6 (30.1-33.3) |
|  | Spain (50-69) | 947 / 7042 | 12.4 (11.5-13.3) | 2419 / 7354 | 31.9 (30.5-33.2) |
|  | | | | | |
| C | Austria (50-74) | 3338 / 6541 | 49.0 (47.5-50.5) | 3537 / 6272 | 55.9 (54.4-57.4) |
|  | Germany (50-74) | 4917 / 9720 | 50.9 (49.8-52.0) | 5127 / 11,428 | 43.8 (42.4-45.2) |
|  | Slovakia (50-74) | 540 / 2128 | 25.9 (24.0-27.9) | 1046 / 2652 | 39.3 (37.4-41.3) |
|  | Latvia (50-74) | 634 / 2671 | 23.1 (21.4-24.7) | 552 / 2114 | 25.8 (23.7-27.8) |
|  | Greece (50-74) | 370 / 3095 | 11.3 (9.9-12.6) | 337 / 3266 | 10.2 (9.0-11.4) |
|  | Iceland (50-74) | 86 / 1446 | 6.0 (4.8-7.3) | 92 / 1496 | 6.1 (4.5-7.6) |
|  | | | | | |
| D | Norway (50-74) | 230 / 3100 | 8.2 (7.1-9.4) | 236 / 3068 | 8.0 (7.0-9.1) |
|  | Poland (50-74) | 552 / 8869 | 6.6 (6.0-7.2) | 578 / 7636 | 7.7 (6.9-8.5) |
|  | Bulgaria (50-74) | 134 / 2523 | 5.7 (4.8-6.7) | 113 / 3178 | 3.6 (2.9-4.3) |
|  | Romania (50-74) | 238 / 6687 | 3.6 (3.1-4.1) | 295 / 6691 | 4.2 (3.7-4.8) |
|  | Cyprus (50-74) | 78 / 1792 | 4.4 (3.4-5.5) | 82 / 2413 | 3.3 (2.5-4.1) |
| **Countries that transitioned between EHIS 2 and EHIS 3** | | | | | |
| A | Denmark (50-74) | 925 / 2711 | 34.8 (32.9-36.6) | 2071 / 3069 | 67.1 (65.4-68.8) |
|  | The Netherlands (55-74) | 234 / 2315 | 10.1 (8.8-11.3) | 1692 / 2562 | 64.5 (62.6-66.5) |
|  | Slovenia (70-74) | 101 / 364 | 26.4 (21.7-31.1) | 330 / 611 | 53.5 (49.4-57.7) |
|  | Lithuania (50-74) | 489 / 2101 | 22.9 (21.1-24.7) | 730 / 1986 | 36.3 (34.2-38.5) |
|  | Belgium (50-74) | 377 / 2284 | 16.4 (14.4-18.5) | 1145 / 3587 | 36.1 (34.0-38.3) |
|  | | | | | |
| B | Czechia (50-74) | 1305 / 3025 | 42.4 (40.3-44.4) | 1603 / 3519 | 44.7 (42.8-46.5) |
|  | Portugal (50-74) | 2004 / 7371 | 35.8 (34.1-37.5) | 2510 / 6542 | 42.2 (40.3-44.2) |
|  | Malta (65-74) | 115 / 691 | 16.3 (13.6-19.1) | 246 / 754 | 33.2 (29.8-36.6) |
|  | Ireland (55-59, 70-74) | 263 / 1440 | 18.1 (16.1-20.1) | 322 / 1224 | 26.5 (23.5-29.6) |
|  | Hungary (50-69) | 231 / 1764 | 13.3 (11.6-14.9) | 277 / 1790 | 15.5 (13.7-17.2) |
|  | | | | | |
| D | Luxembourg (50-74) | 344 / 1412 | 24.2 (21.9-26.5) | 688 / 1714 | 40.6 (38.2-42.9) |
|  | Estonia (50-74) | 125 / 2087 | 6.5 (5.3-7.6) | 420 / 1965 | 21.5 (19.6-23.4) |
|  | Hungary (70-74) | 41 / 320 | 12.9 (9.1-16.6) | 58 / 414 | 14.7 (11.1-18.4) |
|  | Finland (60-69) | 270 / 1339 | 28.2 (27.4-29.0) | 114 / 1257 | 9.4 (7.7-11.1) |

Category A, countries with organized, fully rolled-out programs using fecal tests; Category B, countries with organized fecal test-based programs, partially rolled-out, or with regional coverage only; Category C, countries with opportunistic programs using fecal tests, colonoscopy or flexible sigmoidoscopy; Category D, countries with no program or only a small-scale organized pilot program using fecal tests.

**Table S3**. Changes in utilization of colonoscopy by type of CRC screening offer in 2013-2015 and 2018-2020.

| **Category** | **Country (Age)** | **2013-2015** | | **2018-2020** | |
| --- | --- | --- | --- | --- | --- |
|  |  | **n (test users)/ N (total respondents)** | **Weighted % (95% CI)** | **n (test users)/ N (total respondents)** | **Weighted % (95% CI)** |
| **Countries with stable programs** | | | | | |
| A | Slovenia (50-74) | 702 / 2322 | 29.0 (27.1-31.0) | 1255 / 4051 | 30.1 (28.6-31.6) |
|  | Croatia (50-74) | 286 / 2113 | 14.0 (12.3-15.7) | 406 / 2363 | 17.9 (15.7-20.0) |
|  | | | | | |
| B | Ireland (60-74) | 732 / 2313 | 31.9 (30.0-33.8) | 683 / 1916 | 36.0 (33.4-38.5) |
|  | Italy (50-74) | 1982 / 8897 | 21.7 (20.8-22.6) | 3993 / 17,241 | 22.2 (21.5-22.9) |
|  | Sweden (60-74) | 186 / 1095 | 16.7 (14.5-19.0) | 497 / 2069 | 23.9 (22.0-25.9) |
|  | Spain (50-74) | 1539 / 8462 | 17.6 (16.7-18.6) | 2006 / 9030 | 21.5 (20.5-22.6) |
|  | Malta (55-69) | 188 / 1199 | 15.4 (13.3-17.5) | 272 / 1142 | 23.4 (20.9-25.9) |
|  | | | | | |
| C | Austria (50-74) | 3416 / 6541 | 51.5 (50.1-53.1) | 3390 / 6272 | 54.4 (52.9-55.9) |
|  | Germany (50-74) | 4963 / 9744 | 50.6 (49.5-51.7) | 6486 / 11,724 | 51.7 (50.3-53.1) |
|  | Iceland (50-74) | 602 / 1442 | 41.1 (38.6-43.7) | 783 / 1499 | 51.5 (48.1-54.8) |
|  | Greece (50-74) | 499 / 3114 | 15.3 (13.8-16.8) | 694 / 3309 | 21.4 (19.8-23.1) |
|  | Slovakia (50-74) | 334 / 2128 | 15.1 (13.6-16.7) | 564 / 2662 | 20.9 (19.3-22.6) |
|  | Latvia (50-74) | 298 / 2719 | 10.6 (9.5-11.8) | 334 / 2135 | 16.0 (14.3-17.7) |
|  | | | | | |
| D | Norway (50-74) | 898 / 3120 | 28.0 (26.2-29.7) | 963 / 3078 | 31.4 (29.5-33.2) |
|  | Poland (50-74) | 1382 / 9248 | 15.1 (14.2-16.0) | 1729 / 7920 | 21.9 (20.8-23.1) |
|  | Cyprus (50-74) | 298 / 1792 | 16.2 (14.4-18.0) | 463 / 2413 | 18.8 (17.1-20.5) |
|  | Romania (50-74) | 283 / 6687 | 4.3 (3.7-4.8) | 378 / 6803 | 5.4 (4.8-6.1) |
|  | Bulgaria (50-74) | 56 / 2523 | 2.3 (1.7-2.9) | 132 / 3178 | 4.4 (3.6-5.1) |
| **Countries that transitioned between EHIS 2 and EHIS 3** | | | | | |
| A | Denmark (50-74) | 695 / 2717 | 24.9 (23.3-26.6) | 968 / 3070 | 31.0 (29.3-32.6) |
|  | Belgium (50-74) | 579 / 2263 | 24.4 (22.0-26.8) | 1058 / 3587 | 27.3 (25.4-29.3) |
|  | The Netherlands(55-74) | 499 / 2391 | 20.8 (19.1-22.4) | 708 / 2661 | 26.9 (25.1-28.6) |
|  | Lithuania (50-74) | 337 / 2101 | 15.7 (14.2-17.3) | 532 / 1986 | 26.5 (24.6-28.5) |
|  | | | | | |
| B | Portugal (50-74) | 2258 / 7404 | 35.5 (33.8-37.1) | 2455 / 6622 | 43.4 (41.5-45.3) |
|  | Czechia (50-74) | 761 / 3024 | 24.5 (22.7-26.2) | 979 / 3519 | 27.1 (25.5-28.7) |
|  | Ireland (55-59) | 206 / 946 | 22.2 (19.5-25.0) | 208 / 705 | 26.3 (22.5-30.2) |
|  | Malta (70-74) | 42 / 260 | 16.0 (11.6-20.5) | 86 / 393 | 21.7 (17.6-25.8) |
|  | Hungary (50-74) | 306 / 2093 | 15.2 (13.6-16.8) | 416 / 2313 | 17.7 (16.1-19.4) |
|  | | | | | |
| C | Luxembourg (55-74) | 569 / 1036 | 55.2 (52.0-58.3) | 760 / 1263 | 60.8 (58.0-63.5) |
|  | Luxembourg (50-54) | 133 / 402 | 33.2 (28.6-37.9) | 183 / 476 | 38.5 (34.1-42.9) |
|  | | | | | |
| D | Estonia (50-74) | 255 / 2105 | 12.2 (10.8-13.7) | 363 / 1976 | 19.3 (17.5-21.2) |
|  | Finland (60-74) | 430 / 1770 | 24.2 (22.2-26.2) | 229 / 1965 | 11.4 (9.9-12.8) |

Category A, countries with organized, fully rolled-out programs using fecal tests; Category B, countries with organized fecal test-based programs, partially rolled-out, or with regional coverage only; Category C, countries with opportunistic programs using fecal tests, colonoscopy or flexible sigmoidoscopy; Category D, countries with no program or only a small-scale organized pilot program using fecal tests.

**Table S4**. Changes in utilization of either test by type of CRC screening offer in 2013-2015 and 2018-2020.

| **Category** | **Country (Age)** | **2013-2015** | | **2018-2020** | |
| --- | --- | --- | --- | --- | --- |
|  |  | **n (test users)/ N (total respondents)** | **Weighted % (95% CI)** | **n (test users)/ N (total respondents)** | **Weighted % (95% CI)** |
| **Countries with stable programs** | | | | | |
| A | Slovenia (50-74) | 1537 / 2315 | 65.0 (62.9-67.1) | 2817 / 4036 | 68.0 (66.7-69.7) |
|  | Croatia (50-74) | 601 / 2082 | 29.7 (27.5-31.9) | 865 / 2349 | 37.4 (34.9-40.0) |
|  | | | | | |
| B | Ireland (60-74) | 877 / 2069 | 42.7 (40.6-44.9) | 1013 / 1873 | 55.2 (52.5-57.9) |
|  | Italy (50-74) | 3759 / 8890 | 41.4 (40.3-42.5) | 7974 /17,241 | 45.3 (44.5-46.2) |
|  | Malta (55-69) | 366 / 1198 | 30.6 (27.9-33.3) | 600 / 1142 | 52.5 (49.5-55.4) |
|  | Spain (50-74) | 2219 / 8401 | 25.4 (24.3-26.5) | 3882 / 8926 | 42.6 (41.5-44.1) |
|  | Sweden (60-74) | 311 / 1080 | 28.0 (25.3-30.7) | 827 / 2054 | 38.9 (36.5-41.3) |
|  | | | | | |
| C | Austria (50-74) | 4591 / 6541 | 68.8 (67.3-70.3) | 4570 / 6272 | 72.8 (71.4-74.1) |
|  | Germany (50-74) | 6885 / 9686 | 70.9 (69.9-71.9) | 8153 / 11,403 | 67.9 (66.5-69.3) |
|  | Iceland (50-74) | 616 / 1441 | 42.2 (39.6-44.8) | 791 / 1491 | 52.5 (49.2-56.0) |
|  | Slovakia (50-74) | 703 / 2127 | 33.4 (31.3-35.5) | 1222 / 2648 | 45.6 (43.6-47.6) |
|  | Latvia (50-74) | 778 / 2664 | 28.4 (26.6-30.1) | 717 / 2105 | 33.8 (31.6-36.0) |
|  | Greece (50-74) | 709 / 3074 | 22.7 (20.9-24.5) | 833 / 3246 | 26.1 (24.4-27.9) |
|  | | | | | |
| D | Norway (50-74) | 958 / 3096 | 30.5 (28.7-32.3) | 1019 / 3059 | 33.5 (31.7-35.5) |
|  | Poland (50-74) | 1639 / 8814 | 19.1 (18.2-20.1) | 1891 / 7593 | 24.3 (23.7-26.2) |
|  | Cyprus (50-74) | 329 / 1792 | 17.9 (16.0-19.8) | 494 / 2413 | 20.1 (18.4-21.9) |
|  | Romania (50-74) | 410 / 6687 | 6.3 (5.6-6.9) | 549 / 6682 | 7.9 (7.1-8.7) |
|  | Bulgaria (50-74) | 160 / 2487 | 6.9 (5.8-7.9) | 195 / 3177 | 6.3 (5.4-7.2) |
| **Countries that transitioned between EHIS 2 and EHIS 3** | | | | | |
| A | Denmark (50-74) | 1264 / 2703 | 47.0 (45.1-48.9) | 2302 / 3054 | 74.7 (73.1-76.3) |
|  | The Netherlands(55-74) | 577 / 2297 | 24.9 (23.1-26.7) | 1881 / 2531 | 71.2 (69.4-73.0) |
|  | Belgium (50-74) | 735 / 2171 | 32.7 (30.0-35.4) | 1799 / 3583 | 51.8 (49.4-54.2) |
|  | Lithuania (50-74) | 594 / 2101 | 27.7 (25.8-29.6) | 966 / 1986 | 48.0 (45.8-50.3) |
|  | | | | | |
| B | Portugal (50-74) | 3398 / 7359 | 55.4 (53.7-57.1) | 3813 / 6502 | 65.6 (63.8-67.4) |
|  | Czechia (50-74) | 1634 / 3024 | 53.5 (51.4-55.6) | 2007 / 3518 | 55.9 (54.1-57.8) |
|  | Ireland (55-59) | 220 / 871 | 26.1 (23.1-29.1) | 282 / 691 | 38.3 (33.9-42.7) |
|  | Malta (70-74) | 55 / 259 | 21.0 (16.0-25.9) | 138 / 393 | 35.1 (30.3-39.8) |
|  | Hungary (50-74) | 471 / 2084 | 23.0 (21.1-24.9) | 595 / 2198 | 26.3 (24.3-28.3) |
|  | | | | | |
| C | Luxembourg (55-74) | 620 / 1013 | 61.2 (58.1-64.3) | 925 / 1234 | 75.4 (72.9-77.8) |
|  | Luxembourg (50-54) | 160 / 391 | 41.3 (36.3-46.2) | 223 / 459 | 48.3 (43.7-52.9) |
|  | | | | | |
| D | Estonia (50-74) | 327 / 2086 | 16.1 (14.4-17.7) | 615 / 1964 | 31.9 (29.8-34.1) |
|  | Finland (60-74) | 613 / 1758 | 34.8 (32.6-37.1) | 327 / 1950 | 16.7 (15.0-18.4) |

Category A, countries with organized, fully rolled-out programs using fecal tests; Category B, countries with organized fecal test-based programs, partially rolled-out, or with regional coverage only; Category C, countries with opportunistic programs using fecal tests, colonoscopy or flexible sigmoidoscopy; Category D, countries with no program or only a small-scale organized pilot program using fecal tests.

**Figure S1**. Utilization rate of fecal occult blood test, colonoscopy, and either test by sex for countries in category A

**Figure S2**. Utilization rate of fecal occult blood test, colonoscopy, and either test by sex for countries in category B

**Figure S3**. Utilization rate of fecal occult blood test, colonoscopy, and either test by sex for countries in category C

**Figure S4**. Utilization rate of fecal occult blood test, colonoscopy, and either test by sex for countries in category D

**Figure S5**. Utilization rate of fecal occult blood test, colonoscopy, and either test by age group for countries in category A

**Figure S6**. Utilization rate of fecal occult blood test, colonoscopy, and either test by age group for countries in category B.

**Figure S7**. Utilization rate of fecal occult blood test, colonoscopy, and either test by age group for countries in category C

**Figure S8**. Utilization rate of fecal occult blood test, colonoscopy, and either test by age group for countries in category D

**Figure S9.** Forest plot shows pooled absolute percentage change in use of gFOBT/FIT for each category between stable and transitioned countries. Category A, countries with organized, fully rolled-out programs using fecal tests; Category B, countries with organized fecal test-based programs, partially rolled-out, or with regional coverage only; Category C, countries with opportunistic programs using fecal tests, colonoscopy or flexible sigmoidoscopy; Category D, countries with no program or only a small-scale organized pilot program using fecal tests.


**Figure S10.** Forest plot showing pooled absolute percentage change in use of colonoscopy for each category between stable and transitioned countries. Category A, countries with organized, fully rolled-out programs using fecal tests; Category B, countries with organized fecal test-based programs, partially rolled-out, or with regional coverage only; Category C, countries with opportunistic programs using fecal tests, colonoscopy or flexible sigmoidoscopy; Category D, countries with no program or only a small-scale organized pilot program using fecal tests.

**Figure S11.** Forest plot showing pooled absolute percentage change in use of either test for each category between stable and transitioned countries. Category A, countries with organized, fully rolled-out programs using fecal tests; Category B, countries with organized fecal test-based programs, partially rolled-out, or with regional coverage only; Category C, countries with opportunistic programs using fecal tests, colonoscopy or flexible sigmoidoscopy; Category D, countries with no program or only a small-scale organized pilot program using fecal tests.

**Supplementary reference list (for Table S1)**

34. Tran TN, Peeters M, Hoeck S, Hal GV, Janssens S, De Schutter H*.* Optimizing the colorectal cancer screening programme using faecal immunochemical test (FIT) in Flanders, Belgium from the “interval cancer” perspective. *Br J Cancer* 2022; **126:**1091–99. <https://doi.org/10.1038/s41416-021-01694-2>

35. Belgian Cancer Registry. Contribution of the Belgian cancer registry to screening programs. Available at <https://kankerregister.org/media/docs/academischezitting10.12.15/BCR_PreventionCancerBurden2015_banner.pdf> (Accessed March 17, 2025).

36. United European Gastroenterology. Colorectal cancer screening across Europe. Published March 2019. Available online at ueg.eu/files/779/67d96d458abdef21792e6d8e590244e7.pdf. [Accessed March 18, 2025]

37. Katičić M, Antoljak N, Kujundžić M, et al. Results of National Colorectal Cancer Screening Program in Croatia (2007-2011). *World J Gastroenterol* 2012; **18**:4300–7. doi: 10.3748/wjg.v18.i32.4300.

38. Basu P, Ponti A, Anttila A, et al. Status of implementation and organization of cancer screening in The European Union Member States-Summary results from the second European screening report. *Int. J. Cancer* 2018; **142:** 44–56.

39. Nielsen JB, Berg-Beckhoff G, Leppin A. To do or not to do - a survey study on factors associated with participating in the Danish screening program for colorectal cancer. *BMC Health Serv Res*. 2021; **21**:43. doi: 10.1186/s12913-020-06023-6.

40. Dulskas A, Poskus T, Kildusiene I, et al. National Colorectal Cancer Screening Program in Lithuania: Description of the 5-Year Performance on Population Level. *Cancers (Basel)* 2021; **13:**1129. doi: 10.3390/cancers13051129.

41. Poskus T, Strupas K, Mikalauskas S, et al. Initial results of the National Colorectal Cancer Screening Program in Lithuania. Eur J Cancer Prev. 2015; **24:**76–80. doi: 10.1097/CEJ.0000000000000096.

42. Breekveldt ECH, Toes-Zoutendijk E, van de Schootbrugge-Vandermeer HJ, et al. Factors associated with interval colorectal cancer after negative FIT: Results of two screening rounds in the Dutch FIT-based CRC screening program. *Int J Cancer* 2023; **152**:1536–46. doi:[10.1002/ijc.34373](https://doi.org/10.1002/ijc.34373)

43. Tepeš B, Bracko M, Novak Mlakar D, et al. Results of the FIT-based National Colorectal Cancer Screening Program in Slovenia. *J Clin Gastroenterol* 2017; **51:** e52–e59. doi: 10.1097/MCG.0000000000000662.

44. The Svit prgramme-10 years of colorectal cancer screening in Slovenia: Compendium on the 10th anniversary of the Svit Programme. National Institute of Public Health, Ljublana, 2019. <https://www.program-svit.si/wp-content/uploads/2019/10/SVIT-10-LET-ANG-Elektronska.pdf> (Accessed March 18, 2025)

45. Czech National Cancer Control Programme. Personalised invitations of Czech citizens to cancer screening programmes. Comprehensive Cancer Care Network CanCon. Available at <https://www.onconet.cz/index-en.php?pg=news&aid=987> (Accessed March 2, 2025).

46. Suchanek S, Grega T, Ngo O, et al. How significant is the association between metabolic syndrome and prevalence of colorectal neoplasia? *World J Gastroenterol* 2016; **22**: 8103–11. doi: 10.3748/wjg.v22.i36.8103.

47. Zavoral M, Suchanek S, Majek O, et al. Colorectal cancer screening: 20 years of development and recent progress. *World J Gastroenterol* 2014; **20**: 3825–34. doi: 10.3748/wjg.v20.i14.3825.

48. Rutka M, Molnár T, Bor R, et al. Populációalapú “pilot” colorectalis rákszűrés eredményessége. Csongrád megye, 2015 [Efficacy of the population-based pilot colorectal screening program. Hungary, Csongrád county, 2015]. *Orv Hetil*. 2017; **158:**1658–67. doi: 10.1556/650.2017.30822.

49. Csanádi M, Gini A, de Koning H, et al. Modeling costs and benefits of the organized colorectal cancer screening programme and its potential future improvements in Hungary. *J of Med Screening* 2021; **28:**268–76. doi:10.1177/0969141320968598

50. BowelScreen Programme Report 2016 – 2017 Round Two. Available online at: <https://www.bowelscreen.ie/_fileupload/Programme%20Reports/BowelScreen-Programme-Report%20-2016-2017-FINAL-WEB-21_01_20.pdf> (Accessed March 16, 2025).

51. McFerran E, Kee F, Coleman HG. Colorectal cancer screening: Surely FIT for us too. *Frontline Gastroenterol.* 2019; **10**:445–446. <http://dx.doi.org/10.1136/flgastro-2018-101125>

52. Battisti F, Falini P, Gorini G, et al. Cancer screening programmes in Italy during the COVID-19 pandemic: an update of a nationwide survey on activity volumes and delayed diagnoses. *Ann Ist Super Sanita*. 2022; **58**:16–24. doi: 10.4415/ANN_22_01_03.

53. Zorzi M, Da Re F, Mantellini P, et al. Screening for colorectal cancer in Italy: 2011-2012 survey. *Epidemiol Prev*. 2015; **39**(3 Suppl 1): 93–107.

54. Schreuders EH, Ruco A, Rabeneck L, et al. Colorectal cancer screening: A global overview of existing programmes. *Gut* 2015; **64**:1637–49. <http://dx.doi.org/10.1136/gutjnl-2014-309086>

55. Government of Malta. Colorectal Screening. National Colorectal Cancer Screening Programme. 2020. <https://deputyprimeminister.gov.mt/en/phc/nbs/Pages/Screening-Programmes/Colorectal-Screening.aspx>**.** (Accessed March 17, 2025)

56. Directorate-General of Health, Portugal. Rastreio Oportunístico do Cancro do Cólon e Reto [Opportunistic Screening for Colorectal Cancer]. Lisbon, Portugal, 2014. Available at <https://www.nghd.pt/uploads/noc_rccr_act.pdf> (Accessed March 19, 2025).

57. Nogueira RA. (Programa Nacional para as Doenças Oncológicas: o despacho n.º 8254/2017, de 21 de setembro, do Secretário de Estado Adjunto e da Saúde, merece mais atenção e representa um primeiro passo para a orientação de soluções)National Program for Oncological Diseases: The Order No. 8254/2017 of September 21, of the Assistant Secretary of State and Health, deserves more attention and represents a first step towards the guidance of solutions. *Portuguese J of Gen and Fam Med*. **34**:104–9. <https://doi.org/10.32385/rpmgf.v34i2.12403>

58. Currais P, Mão de Ferro S, Areia M, Marques I, Mayer A, Dias Pereira A. Should colorectal cancer screening in Portugal start at the age of 45 years? A cost-utility analysis. *GE Port J Gastroenterol.* 2021; **28**:311–18. doi: 10.1159/000513592.

59. Trejo DS, Villares IP, Pinol JAE, et al. Implementation of colorectal cancer screening in Spain: Main results 2006-2011. *Eur. J. Cancer Prev*. 2017; **26**:17–26.

60. Network of Cancer Screening Programmes. Evaluación programas de cribado de cancer colorrectal-2017. (2018). Available at [file:///C:/Users/i200a/Downloads/programasdeccr2017.pdf](file:///C:\Users\i200a\Downloads\programasdeccr2017.pdf) pg. 5-13. (Accessed March 18, 2025).

61. Senore C, Basu P, Anttila A, et al. Performance of colorectal cancer screening in the European Union Member States: Data from the second European screening report. *Gut* 2019; **68:**1232–44.

62. Lund University Cancer Center. Screening for colorectal cancer starts this spring. Available online at <https://www.lucc.lu.se/article/screening-colorectal-cancer-starts-spring#:~:text=In%20May%2C%20screening%20for%20colorectal%20cancer%20will%20be,lives%20are%20expected%20to%20be%20saved%20per%20year>. (Accessed March 19, 2025)

63. Gsur A, Baierl A, Brezina S. Colorectal Cancer Study of Austria (CORSA): A Population-Based Multicenter Study. *Biology* 2021; **10:**722. https://doi.org/10.3390/ biology10080722

64. Program for the early detection of colorectal cancer. Available at <https://www.g-ba.de/themen/methodenbewertung/ambulant/frueherkennung-krankheiten/erwachsene/krebsfrueherkennung/darmkrebs-screening/> (Accessed March 12, 2025).

65. Heisser T, Weigl K, Hoffmeister M, Brenner H. Age-specific sequence of colorectal cancer screening options in Germany: A model-based critical evaluation. *PLoS Med.* 2020; **17**: e1003194. doi: 10.1371/journal.pmed.1003194.

66. Mārcis Leja. Organised colorectal cancer screening needed in Latvia. (Presentation at the Cancer Control Joint Action 2014: Available at <https://www.cancercontrol.eu/archived/news/10/26/Organised-colorectal-cancer-screening-needed-in-Latvia/d%2cnews.html> (Accessed March 17, 2025).

67. Ricová JT. Súčasný skríning kolorektálneho karcinómu na Slovensku a úloha VLD. (2020). Available online (in Slovak) <https://www.noisk.sk/files/2021/2021-02-10-skrining-kolorektalneho-karcinomu-na-slovensku-a-uloha-vld.pdf> [Accessed March 14, 2025].

68. Tsvetanova DR, Dimitrova DD, Angelova LB, et al. Feasibility of immunochemical faecal occult blood testing for colorectal cancer screening in Bulgaria. *J BUON*. 2015; **20:**413–20.

69. OECD (2023), *EU Country Cancer Profile: Cyprus 2023*, EU Country Cancer Profiles, OECD Publishing, Paris, <https://doi.org/10.1787/86732eb6-en>.

70. Innos K, Reima H, Baburin A, Paapsi K, Aareleid T, Soplepmann J. Subsite- and stage-specific colorectal cancer trends in Estonia prior to implementation of screening. *Cancer Epidemiol.* 2018; **52**:112–19. doi: 10.1016/j.canep.2017.12.016.

71. Reima H, Soplepmann J, Elme A, et al. Changes in the quality of care of colorectal cancer in Estonia: a population-based high-resolution study. *BMJ Open.* 2020; **10**: e035556. doi: 10.1136/bmjopen-2019-035556.

72. Färkkilä M, Heinävaara S, Hyöty M, et al. Protocol for colorectal cancer screening. Recommendation of the expert group set up by the National Cancer Screening Steering Group. 2021. <https://syoparekisteri.fi/assets/files/2021/11/Protocol-for-and-tests-used-in-colorectal-cancer-screening.pdf> (Accessed March 10, 2025)

73. Guðlaugsdóttir S. Implementing colorectal cancer screening program in Iceland. In: WEO Barcelona October 2015 proceedings of WEO Colorectal Cancer Screening Meeting. Barcelona, Spain. Available from: <https://www.worldendo.org/wp-content/uploads/2016/08/6_sunna_guolaugsdottir_iceland_ueg2015.pdf>

74. Ministry of Health of Luxemboug (Grand-Duché de Luxembourg). Programme de Dépistage Organisé du Cancer ColoRectal Grand-Duché de Luxembourg 2022. Available at [file:///C:/Users/i200a/Downloads/pdoccr-programme-de-depistage.pdf](file:///C:\Users\i200a\Downloads\pdoccr-programme-de-depistage.pdf) (Accessed March 12, 2025).

75. Bhargava S, Czapka E, Hofvind S, Kristiansen M, Diaz E, Berstad P. Polish immigrants' access to colorectal cancer screening in Norway - a qualitative study. *BMC Health Serv Res.* 2022; **22**:1332. doi: 10.1186/s12913-022-08719-3.

76. Cancer Registry of Norway. Colorectal Cancer Screening, Bowel Cancer Screening in Norway – A Pilot Study. Available online: <https://www.kreftregisteret.no/en/screening/Screening-for-colorectal-cancer/> (Accessed March 12, 2025).

# 77**.** Giske Ursin. Pilot Study of a National Screening Programme for Bowel Cancer in Norway. ClinialTrials.gov. 2012. <https://beta.clinicaltrials.gov/study/NCT01538550> (accessed on March 12, 2025**).**

78. Randel KR, Schult AL, Botteri E, et al. Colorectal cancer screening with repeated fecal immunochemical test versus sigmoidoscopy: Baseline results from a randomized trial. *Gastroenterology*. 2021; **160**:1085–96.e5. doi:10.1053/j.gastro.2020.11.037

79. Krzeczewski B, Hassan C, Krzeczewska O, et al. Cost-effectiveness of colonoscopy in an organized screening program. *Pol Arch Intern Med.* 2021; **131**:128–35. doi:10.20452/pamw.15779

80. Wieszczy P, Kaminski MF, Franczyk R, et al. Colorectal Cancer Incidence and Mortality After Removal of Adenomas During Screening Colonoscopies. *Gastroenterology* 2020; **158**:875–83.e5. doi: 10.1053/j.gastro.2019.09.011.

81. Polish Society of Oncology. Cancer control strategy for Poland 2015-2024. (2017). Available online at <https://ligawalkizrakiem.pl/images/content/Strategia-Walki-z-Rakiem-w-Polsce/Strategia_wersja_ang_2017.pdf> (Accessed March 17, 2025).

82. Bărbulescu LN, Mogoantă SȘ, Bărbulescu LF, Kamal C, Popa DL, Popa RT. A pilot colorectal cancer study using fecal occult blood tests and colonoscopy to identify the weaknesses of the Romanian public healthcare system before implementing national screening. *Int. J of Envir Res and Public Health* 2023; **20**:2531. <https://doi.org/10.3390/ijerph20032531>
